# Deep Neural Network-Based Semantic Segmentation of Microvascular Decompression Images

**DOI:** 10.3390/s21041167

**Published:** 2021-02-07

**Authors:** Ruifeng Bai, Shan Jiang, Haijiang Sun, Yifan Yang, Guiju Li

**Affiliations:** 1 Changchun Institute of Optics, Fine Mechanics and Physics, Chinese Academy of Sciences, Changchun 130033, China; bairuifeng18@mails.ucas.ac.cn (R.B.); sunhj@ciomp.ac.cn (H.S.); yangyifan17@mails.ucas.ac.cn (Y.Y.); liguiju@ciomp.ac.cn (G.L.); 2 University of Chinese Academy of Sciences, Beijing 100049, China

**Keywords:** microvascular decompression image, semantic segmentation, DeepLabv3+, encoder structure, decoder structure

## Abstract

Image semantic segmentation has been applied more and more widely in the fields of satellite remote sensing, medical treatment, intelligent transportation, and virtual reality. However, in the medical field, the study of cerebral vessel and cranial nerve segmentation based on true-color medical images is in urgent need and has good research and development prospects. We have extended the current state-of-the-art semantic-segmentation network DeepLabv3+ and used it as the basic framework. First, the feature distillation block (FDB) was introduced into the encoder structure to refine the extracted features. In addition, the atrous spatial pyramid pooling (ASPP) module was added to the decoder structure to enhance the retention of feature and boundary information. The proposed model was trained by fine tuning and optimizing the relevant parameters. Experimental results show that the encoder structure has better performance in feature refinement processing, improving target boundary segmentation precision, and retaining more feature information. Our method has a segmentation accuracy of 75.73%, which is 3% better than DeepLabv3+.

## 1. Introduction

At present, deep neural networks have been widely introduced into the research of biomedical image classification [1,2,3,4], segmentation [5,6], identification [7,8], brain research [9] and other fields, and have achieved remarkable results. In terms of segmentation, image semantic segmentation is the focus of digital image processing and machine-vision research, and a simple high-performance approach to semantic segmentation is proposed by Csurka et al. [10]. Each pixel in the image is classified according to its category and the prediction containing “semantic” information [11]. Image semantic segmentation involves the research and application of virtual reality, industry, civil, medical and other fields and has achieved remarkable results. In the medical field, cerebrovascular images are generally obtained by computed tomography angiography (CTA), digital subtraction angiography (DSA), magnetic resonance angiography (MRA), etc., and processed according to the traditional algorithm and the method of deep learning. However, the processing of true-color microvascular decompression images is deficient, and the acquisition of true-color medical images is more convenient to compare with traditional medical images. The segmentation of cerebral vessels and cranial nerves from microvascular decompression images has become an important research direction in the future development of intelligent medical treatment. The ultimate goal is to reduce the stress of surgeons, improve the speed of surgery, reduce the negative injuries and complications of surgery, allow general doctors to achieve an expert level of cognition, and allow experts to operate more efficiently.

Blood-vessel segmentation based on traditional methods includes matched filtering [12,13,14], multiscale approaches [15,16,17,18,19,20], a morphology-based approach [21], an active contour model [22,23,24,25,26,27], level set approaches [28,29,30], region growing [31,32,33,34], and region merging [35]. Research on the semantic segmentation of cerebrovascular images based on deep learning requires the collection and annotation of cerebrovascular images. Special equipment is used to solve the collection problem of cerebrovascular images. The data annotation needs to be done manually. The image semantic-segmentation method based on deep learning is mainly divided into image semantic segmentation based on the regional classification (ISSbRC) and image semantic segmentation based on the pixel classification (ISSbPC). ISSbRC has the disadvantages of low segmentation accuracy, slow segmentation speed, and low computational efficiency, which entails the existence of ISSbPC. First, Long et al. [36] proposed a fully convolutional network (FCN) that is compatible with images of any size and uses fully-supervised learning for image semantic segmentation. FCN is improved on the basis of Visual Geometry Group VGG-16 [37]. The full connection layer in convolutional neural network (CNN) [38] is replaced by the convolutional layer, and the skip layer method is used to combine the feature map generated by the intermediate convolutional layer. The use of the skip layer is conducive to the fusion (concatenation and addition) of deep rough features and shallow fine features. Then, bilinear interpolation is used for upsampling to predict the classification of each pixel, and the rough-segmentation results are converted into fine-segmentation results. Because the pooling operation reduces the resolution of the feature map, Ronneberger et al. [39] proposed a network model of the encoder and decoder structure, U-Net, which performs downsampling during the encoder process to gradually reduce the resolution of the feature map. In the decoder process, upsampling is performed to gradually restore object details and image resolution. 

Another network-model structure is the SegNet [40]. The SegNet network calculates the classification of each pixel based on the prior probability. The encoder is composed of a fully convolutional network and is downsampled and decoded through operations such as convolutional pooling. The encoder is composed of deconvolution, and it upsamples its input according to the transmission index of the encoder. Deconvolution is used to restore detailed information and corresponding spatial dimensions. The encoder and decoder structure avoids the problem that the resolution of feature map decreases after pooling operation and restores the spatial dimension and pixel position information of the image. Nasr-Esfahani et al. [41] proposed a basic CNN for the segmentation of vessels in coronary angiogram, but the results were not significant. Phellan et al. [42] explored a relatively shallow neural network in MRA images, which was the first application of convolutional neural networks to solve the problem of cerebrovascular segmentation. However, due to the small sample size and shallow network, the performance was limited. Mo et al. [43] proposed a multilevel FCN with deep supervision. Although it segmented the thick vessels properly, most of the fine vessels and microvessels were missed. Jiang et al. [44] proposed that transfer learning in FCN could complete the segmentation of vascular structure, but it could not perform robust segmentation of vascular regions. Noh et al. [45] proposed the scale-space approximated CNN, which retained the receptive field, increased the network depth, and showed excellent performance in the segmentation of blood vessels. However, the elimination of the downsampling layers had a negative impact on some datasets. Livne et al. [46] used a network-based encoder and decoder structure to segment the cerebral vessels in MRI images. The encoder–decoder U-Net architecture captures contextual information and transfers it to the higher-resolution layers, but it could not accumulate more features or handle details such as fine blood vessels.

Due to the shortage of the networks selected in the above research, we chose the DeepLabv3+ [47] as the basic framework, which has a good performance in image semantic segmentation. The true-color image dataset obtained by the microscope imaging device was taken as the experimental dataset. This network model has great challenges in medical image segmentation, and it has shortcomings such as incorrect classification, inaccurate target edge segmentation, and lack of target details. By referring to the idea of the encoder and decoder structure, and further analyzing the original image, it is found that it is difficult to segment the edge of the cerebral vessels. First, the DeepLabv3+ encoder structure was improved by introducing a feature distillation block (FDB) into the backbone network to refine the feature. Then, the decoder structure was improved to fuse the feature information of the bottom layer and the top layer as much as possible. By introducing the atrous space pyramid pooling (ASPP) module [48] into the decoder structure, a complete segmentation with clear edges was obtained. The experimental results show that our method can segment and identify the cerebral vessels and cranial nerves from microvascular decompression images and certainly contribute to future intelligent medical treatment.

## 2. Related Work

Chen et al. [49] proposed the DeepLab to improve the shortcomings of FCN, such as lack of spatial consistency and imprecise segmentation. The network used a fully connected conditional random field (FCCRF) to obtain a coarse-segmentation map. The DeepLab performed boundary optimization and used atrous convolution to expand the receptive field of the feature map to complete semantic segmentation. The DeepLabv2 [50] and the ASPP module were proposed based on the DeepLab. The ASPP module integrates multiscale features, increases the receptive field, and improves the segmentation accuracy. Based on DeepLab and DeepLabv2 ideas, DeepLabv3 [51] was put forward, which improved the ASPP module by introducing batch normalization (BN) and removing FCCRF. Because DeepLabv3 used a pooling operation, the detailed information of the target boundary was lost, and the dilated convolution calculation was relatively large. DeepLabv3+ was later introduced, performing better than DeepLabv1, v2, and v3, making use of depth-wise separable convolution. DeepLabv3 was used as an encoder and a decoder had to be added to restore target boundary details. In DeepLabv3+, the lightweight Xception [52] is first used for feature extraction, and then the ASPP module helps to obtain multiscale feature information. The obtained multiscale feature information is processed by the 1 × 1 convolution. After four times of upsampling, it is concatenated with the 1 × 1 convolution processing features of the backbone network. Then, the 3 × 3 convolution fine-tuning feature is used, and upsampling is performed four times again to obtain the final prediction image.

DeepLabv3+ performs well on the dataset commonly used in semantic segmentation, performing at 89.0% and 82.1% with the PASCAL VOC2012 and Cityscapes dataset, respectively [47]. In this article, DeepLabv3+ is used to perform semantic segmentation of microvascular decompression images. DeepLabv3+ uses the microvascular-decompression-image dataset for training. The experimental results show that the semantic segmentation of microvascular decompression images is not ideal. The method has the problem of target pixel mixing and also contains various other shortcomings, such as blurry target boundary segmentation, incomplete contour, and insufficient feature information.

## 3. Model

**Figure 1:**
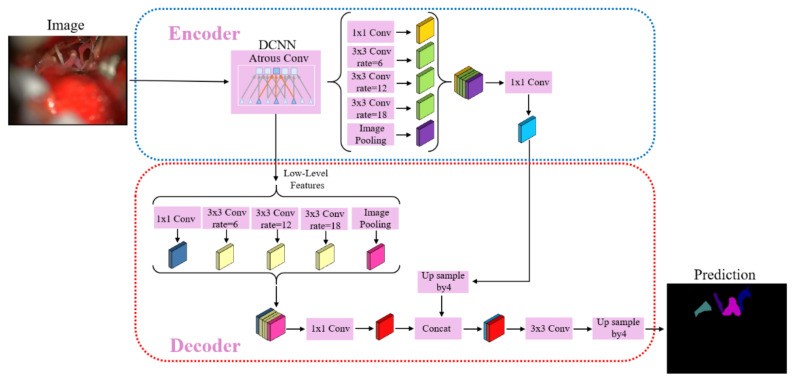
The structure of the semantic-segmentation network for microvascular decompression images.

To cure the problems of DeepLabv3+ in the semantic segmentation of microvascular decompression image dataset, our method improves the encoder and decoder structure of DeepLabv3+. First, the second depth-wise separable convolution is replaced by the feature distillation block (FDB) in the backbone network. Secondly, the decoder structure is optimized, and the ASPP module is added to obtain more feature information. We will elaborate on the details of the improvement in the following section. The improved network model is shown in Figure 1.

In the proposed network model, the backbone network selects Xception_65 with 65 network layers. Xception_65 adopts depth-wise separable convolution to realize feature extraction. In the backbone network, features are distilled to obtain more refined features. Then, the feature maps obtained from the backbone network are input into the ASPP module, and after a feature extraction at different sampling rates is conducted, the multiscale context information is finally effectively captured. The ASPP module is composed of a 1 × 1 standard convolution, three 3 × 3 dilated convolution with sampling rates of 6, 12 and 18, and global average pooling. Each convolution kernel has 256 and batch normalization layer. Finally, all feature maps are concatenated by a 1 × 1 convolution.

The features obtained by the ASPP module are upsampled four times by bilinear interpolation to obtain the enlarged feature map. The low-level features obtained from the second convolutional layer in the backbone network and the low-level features obtained from the first block are also mapped to the ASPP module of the decoder, which has the same structure as the ASPP module in the encoder. After that, the high-level and low-level feature maps are concatenated and the features are fine-tuned through the 3 × 3 convolution. Finally, using the bilinear interpolation method to upsample four times, the final segmentation map is obtained.

The loss function used in this article is the crossentropy loss function [53]. The loss function formula:

(1) $$L=-\sum_{i=1}^{N}y^{\left(i\right)}\log{\overset{⌢}{y}}^{\left(i\right)}+(1-y^{\left(i\right)})\log(1-\log{\overset{⌢}{y}}^{\left(i\right)})$$

where *L* is the training loss, *N* is the number of samples, *y* is the actual sample label, and $\overset{⏜}{y}$ is the predicted label. *y* takes values 0 or 1, and $\overset{⏜}{y}$ takes the value of (0,1). The smaller the *L* value, the more accurate the prediction result and the better the performance of the network model.

### 3.1. Network Backbone

The Xception_65 is a lightweight network based on inception that is composed of a depth-wise separable convolution and residual network [54]. Standard convolution extracts all spatial information and channel information; the Xception_65 extracts the information separately to achieve better results. The Xception_65 consists of an entry flow that contains 11 convs, a middle flow that contains 48 convs, and an exit flow that contains 6 convs, with a total of 65 layers. The Xception_65 network has an excellent performance that reduces the computational complexity, accelerates the model’s training process, and ensures the model’s learning ability.

We have made further improvements to the Xception_65. In 2018, Hui et al. [55] proposed an information distillation network (IDN), which divides the intermediate features into two parts along the channel dimension: one part is retained, and the other part is processed through subsequent convolutional layers. By using this channel segmentation strategy, IDN can aggregate partially-retained local short-path information with current information and obtain a good performance in feature extraction. In 2019, Hui et al. [56] improved the IDN and designed an information multidistillation block (IMDB) to extract features at a fine-grained level. In 2020, Liu et al. [57] improved the information multidistillation network (IMDN) and proposed the lightweight and accurate residual feature distillation network (RFDN). The shallow residual block (SRB) was proposed as the basic block of RFDN to maximize the benefits of residual learning while maintaining the network’s lightness.

**Figure 2:**
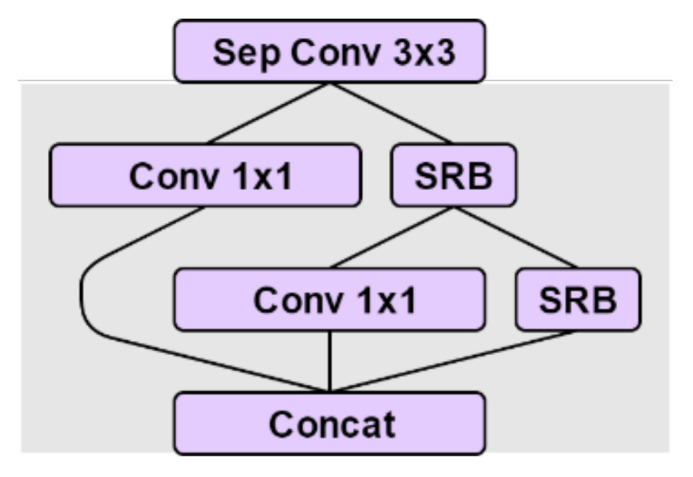
FDB (feature distillation block).

The FDB uses multiple feature connections to learn more discriminative features, as shown in Figure 2. The SRB, which is the main building block of the FDB, enables the network to maximize the benefits of residual learning while maintaining sufficient lightness. The SRB consists of one convolution layer, an identical connection, and an activation unit at the end. Compared with ordinary convolution, the SRB can benefit from residual learning without introducing additional parameters [54]. It is easy to combine the SRB with feature extraction connections to build a better performance network.

**Figure 3:**
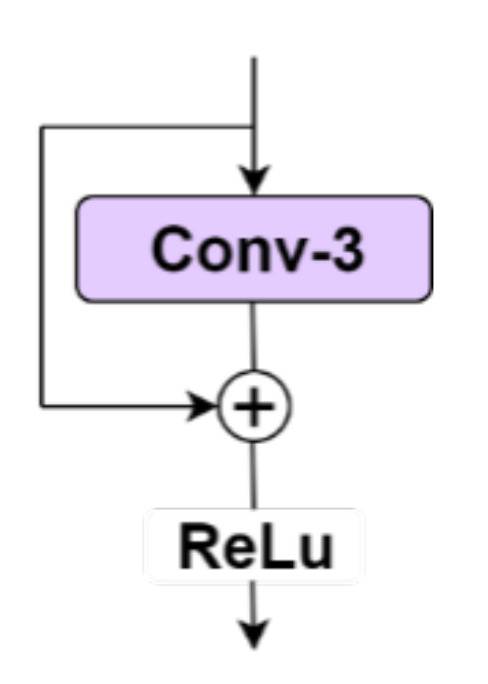
SRB (shallow residual block).

**Figure 4:**
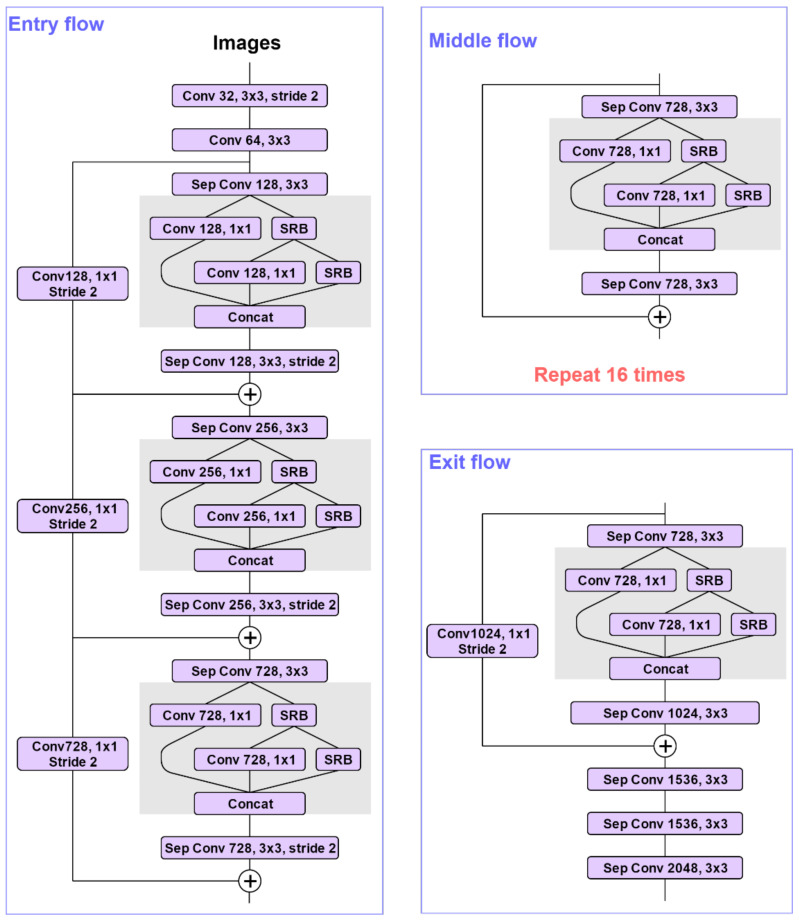
The Xception model is modified as follows: The second depth-wise separable convolution is replaced by the FDB in the residual block of input flow, middle flow, and exit flow.

In the FDB, using the 1 × 1 convolution for channel reduction is more effective than in a lot of other CNN models. The convolution of 1 × 1 has greatly reduced the number of arguments and introduces the SRB, as shown in Figure 3. The FDB is located on the body of Xception_65, as shown in Figure 4, which not only considers the spatial context but also has a good refinement feature.

### 3.2. Decoder Structure Optimization

**Figure 5:**
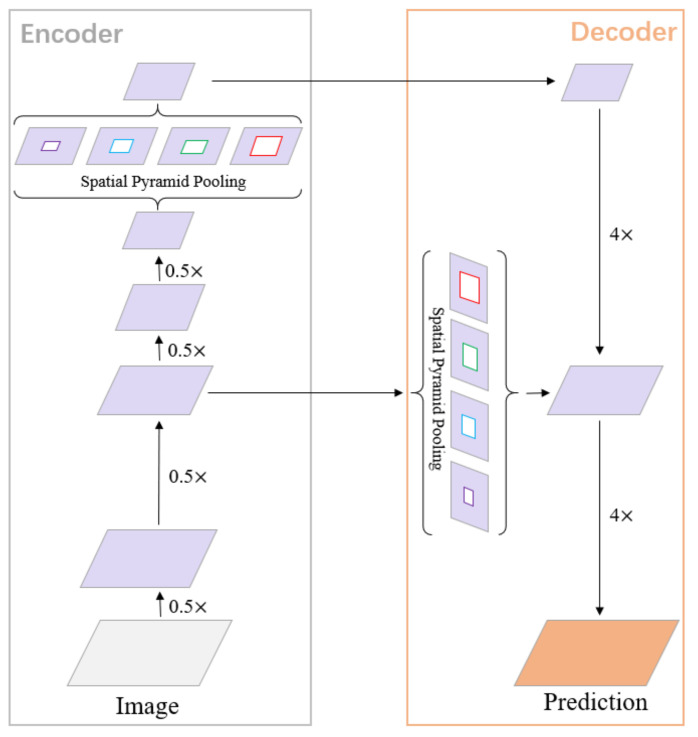
Vertical structure of semantic segmentation of microvascular decompression images.

Figure 5 shows the improved DeepLabv3+ encoder and decoder structure, with the encoder structure on the left and the decoder structure on the right.

Under the DeepLabv3+ encoder and decoder structure, the semantic segmentation of microvascular decompression image cannot distinctly determine the target boundary. The decoder structure directly upsampling four times causes some feature information to be lost. Therefore, we added the ASPP module in the decoder structure. The low-level feature, which is processed by ASPP in the decoder structure, and the high-level-feature map, which is upsampled four times, in the encoder structure are concatenated. This makes the segmentation-boundary information more complete and the semantic information clearer.

## 4. Experiments

A self-made training set is used under our method and we tested the test set with the trained network model. Compared to the other advanced semantic-segmentation methods, our method has better segmentation accuracy. We have also experimentally shown which improvements are more effective.

### 4.1. Dataset

At present, there are few public datasets of medical images available, and the annotation of medical images requires professional evaluation, which makes labeled datasets even scarcer. The situation is even more severe in the fields of semantic segmentation of microvascular decompression images. Here, we actively cooperated with the First Hospital of Jilin University. Experts used Labelme annotation tools to manually label, and then original images and annotation files were generated in the same format as the PASVOL VOC 2012 dataset. In the self-designed dataset, there are 1993 RGB microvascular decompression images and the corresponding well-labeled masks for microvascular decompression-image segmentation. The image sizes are 768 × 576 and 1920 × 1080. The dataset has nine categories (when a background category is numbered 0, there are 10 categories that are added). The names of the categories and their corresponding colors are shown in Table 1.

In Table 1, “cn5” means the trigeminal nerve, “cn7” indicates the facial nerve, “cn9” represents the glossopharyngeal nerve, “cn10” means the vagus nerve, “aica” presents the anterior inferior cerebellar artery, “pica” means the posterior inferior cerebellar artery, “aica + cn7” describes the anterior inferior cerebellar artery and the facial nerve, “pica + cn7” presents the posterior inferior cerebellar artery and the facial nerve, and “pv” means the petrosal vein.

The dataset has 1993 images in the experiment. A training set of 1819 images and a test set of 174 images were randomly selected from the dataset. We got images of different sizes. For the convenience of training, we set the size of the image to 512 × 512.

### 4.2. Pre-Processing

**Figure 6:**
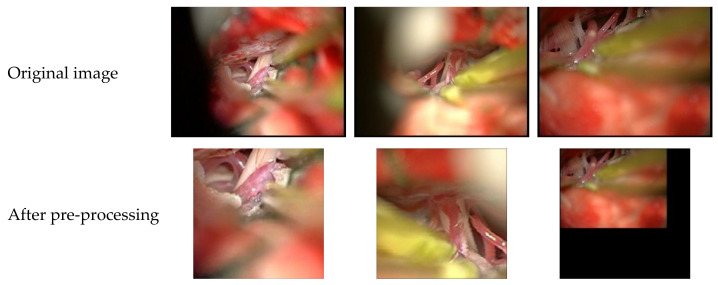
Examples of pre-processing.

Research shows that the number of training samples is important to the performance of deep neural networks. For a small dataset, artificial data augmentation is a common approach to generate sufficient training samples. Due to the limited size of the microvascular decompression-image dataset, this study uses random horizontal flip, random scale cropping, random Gaussian blur, and normalization strategies for data augmentation, as shown in Figure 6.

### 4.3. Network Training

The experimental environment is Intel(R)Core™i7-9700K CPU@3.60GHz, Ubuntu 18.04, 64-bit operating system, running memory 32G, NVIDIA GEFORCE RTX 2080Ti, CUDA10.1, CuDNN7.6.0, and Python3.7.

The improved DeepLabv3+ network model is trained using the microvascular decompression-image dataset. The training parameters are shown in Table 2.

In Table 2, “num clones” means the number of GPUs during the training; “iterations” indicates the number of iterations; “atrous rate” means the dilated convolution rate in the ASPP module during the training; “output stride” represents the output stride of the encoder structure; “decoder output stride” means the output stride of the decoder structure; “crop size” indicates the size of the image; and “batch size” means the number of images read in a batch.

**Figure 7:**
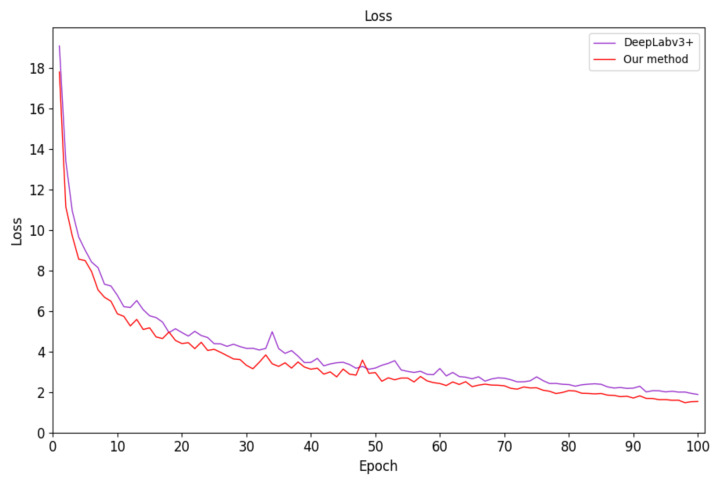
Network-model-training loss curve.

**Figure 8:**
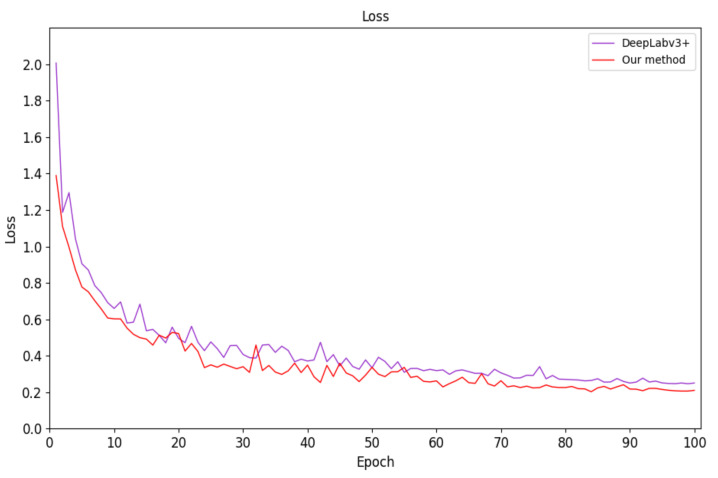
Network-model-validation loss curve.

The proposed method takes approximately 3 h per 10,000 iterations. In the same experimental environment, we trained U-Net, PSPNet [58], DeepLabv3+, DANet [59], and FastFCN [60] using the microvascular decompression image dataset. The corresponding semantic-segmentation model was obtained and compared to the test set using our method. Figure 7 and Figure 8 show the average loss curve of the improved network model and DeepLabv3+ during training and validation. It can be seen from the Figure 7 and Figure 8 that in the initial training stage, the loss decreases rapidly but gradually becomes stable as the number of training iterations increases. Furthermore, the loss reduction of our method is better than in DeepLabv3+.

### 4.4. Analysis of Results

**Figure 9:**
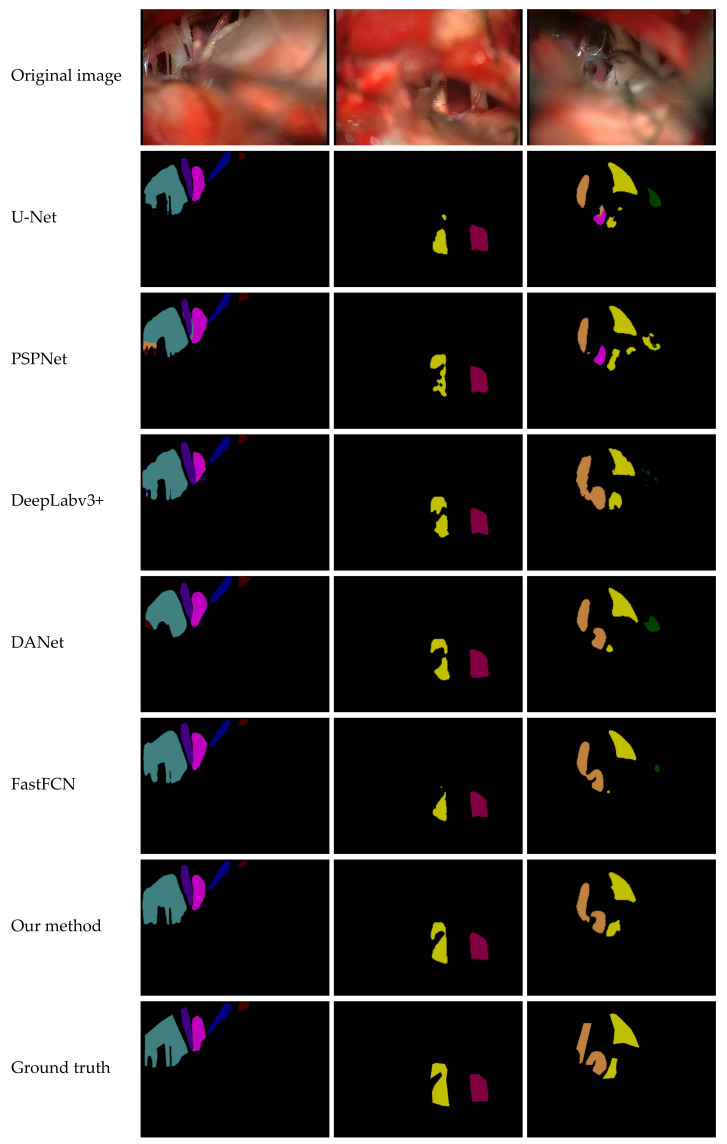
Experimental results and comparison with other methods.

The test set was inputted into the trained semantic-segmentation models U-Net, PSPNe, DeepLabv3+, DANet, and FastFCN, and we compared the results. As shown in Figure 9, from top to bottom, there are the original image, U-Net, PSPNet, DeepLabv3+, DANet, and FastFCN, as well as our method, and the ground truth images.

As can be seen from the experimental results in Figure 9, in the first column, U-Net, PSPNet, DeepLabv3+, DANet, and FastFCN do not accurately locate the segmentation boundary of “cn10”and the object contour is not clear. Furthermore, there are obvious multipixel mixing problems in the PSPNet and DANet methods. The “cn5” in the second column is incomplete in the boundary-segmentation methods of U-Net, PSPNet, DeepLabv3+, DANet, and FastFCN, showing obvious missing target contour segmentation. In the third column, the U-Net, DeepLabv3+, DANet, and FastFCN methods are incorrectly segmented. Segmenting the extra “cn7”, there is a multipixel mixing in the U-Net method, and the “pv” and “pica” classifications are incorrect in PSPNet. The target contour segmentation proposed in this article is more complete and contains more feature information.

However, the segmentation of “cn10” and “pv” is incomplete in these methods. The segmentation results of “cn10” in the first column and “pv” in the third column are quite different from the actual situation. Moreover, compared with other methods, the segmentation results obtained in our method are the closest to the ground truth. Our method can obtain the segmentation results with more feature information, which are closer to the actual situation.

#### 4.4.1. Analysis and Comparison of Test Data

Mean intersection over union (MIoU) of the network model trained by U-Net, PSPNet, DeepLabv3+, DANet, FastFCN, and our method was tested by the test set. The MIoU value is an important indicator to measure the accuracy of image segmentation. MIoU is accumulated after calculating the IoU values of each category and then averaging them. IoU indicates the overlap ratio between the generated prediction area and the ground truth. The ratio is their intersection to the union. The ideal situation is a full overlap, with the ratio being one.

The higher the MIoU value, the more accurate the segmentation result and the better the performance of the network model. MIoU is calculated as follows:

(2) $$MIoU=\frac{1}{k+1}\sum_{i=0}^{k}\frac{p_{ii}}{\sum_{j=0}^{k}p_{ij}+\sum_{j=0}^{k}p_{ji}-p_{ii}}$$

In the above formula, k represents the number of categories. If the background is included, there are k+1 categories. i represents the true value, and j represents the predicted value. $p_{ii}$ represents the total number of pixels whose category is correctly classified as i. $p_{ij}$ represents the total number of pixels whose category i is predicted as j, and $p_{ji}$ vice versa. $p_{ij}$ and $p_{ji}$ represent pixels that are misclassified.

The test set was used to test the MIoU values of DeepLabv3+ and the proposed network after training. Our training output stride was 16, and the test output stride was 16. The test results are shown in Table 3.

In Table 3, Train OS indicates the output stride during training, and Eval OS means the output stride during evaluation.

In this article, Train OS of 16 and Eval OS of 16 were selected, and compared with the current advanced segmentation models U-Net, PSPNet, DANet, and FastFCN. After training these segmentation models with the training set, we used the test set to calculate the MIoU value of the trained network model. The final precision value of semantic segmentation is shown in Table 4.

It can be seen from Table 4 that our method obtains the highest segmentation accuracy value compared to the other methods.

#### 4.4.2. Improved Module Validity Verification

In order to further verify the effectiveness of the FDB and optimized decoder structure, we tested them separately, as shown in Table 5.

The check marks in Table 5 indicate the presence of a certain module. “Encoder” represents the encoder structure in DeepLabv3+, and “Decoder” represents the decoder structure in DeepLabv3+. “Our Encoder” represents our improved encoder structure, “Our Decoder” represents our improved decoder structure. The second row in Table 5 refers to the original DeepLabv3+ decoder structure is replaced with the optimized decoder structure. The corresponding MIoU value is subsequently calculated. The second row in Table 5 refers to the original DeepLabv3+ encoder structure replaced with the optimized decoder structure. The third row in Table 5 represents the improved network model of DeepLabv3+. During the test, the training-output stride and the test-output stride were set to 16.

Table 5 shows that both methods have certain improvements compared to the original DeepLabv3+, and the accuracy of image semantic segmentation is improved to different degrees. Compared to the optimized encoder structure, the optimized decoder structure has a relatively greater impact on the semantic-segmentation results.

**Figure 10:**
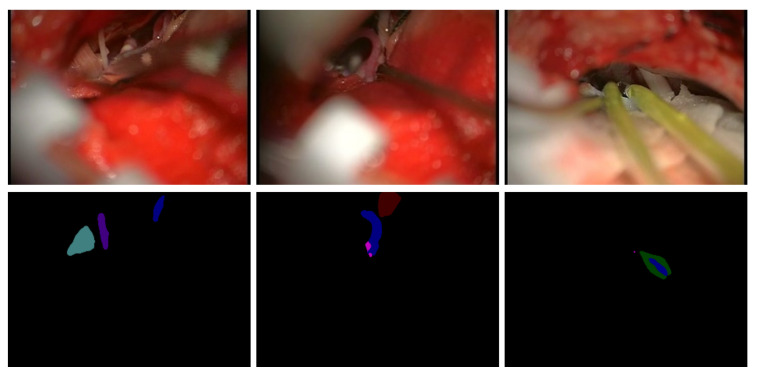
Failure case.

Figure 10 shows some failed cases of the semantic-segmented network model. The first row in the figure shows the original image, and the second row shows the experimental results obtained by the proposed method, which indicates that there are errors in the segmentation of cerebral vessels and cranial nerves. In the first image, “pica” is unsegmented. The second image has the problem of multipixel mixing. The third image has the problems of incorrect segmentation and multipixel mixing.

## 5. Discussion

For microvascular decompression, the changes of brain tissue and structure are dynamic during surgery, and the release of cerebrospinal fluid occurs randomly. The tissue is stretched to cause deformation, or the tissue is removed and collapsed, and cerebral vessels and cranial nerves are displaced. The surgeon needs to identify the structure and estimate the position through experience, which leads to many uncertainties in surgery and the occurrence of surgical risks, and in severe cases, can even cause disability and death. Our method has a remarkable effect on the segmentation of cerebral vessels and cranial nerves under the condition of brain-tissue deformation and drift. Furthermore, the collected semantic information of various cerebral vessels and cranial nerves is correct, and the classification is accurate. To a certain extent, the performance of semantic segmentation of microvascular decompression images is improved. Our method improves the decision and judgment of the surgeon and reduces the uncertainty and risk of surgery. However, the method also suffers from some drawbacks. Due to the blurring of the edges between the brain tissue and the cerebral vessels and cranial nerves, the edges of the segmentation are not detailed enough. In addition, the similarities between different types of cerebral vessels and different types of cranial nerves also lead to inaccurate partial segmentation. Our future research is focused on solving the above problems and further improving the performance of semantic segmentation.

## 6. Conclusions

We propose a semantic-segmentation model of microvascular decompression images based on the improved DeepLabv3+. In this model, the FDB is added in the encoder structure, and the ASPP module is added in the decoder structure, so as to improve the performance of semantic segmentation of microvascular decompression images. The existing methods in processing the semantic segmentation of microvascular decompression images rely on large medical equipment to obtain the cerebrovascular images. This article deals with the more convenient true-color images. The existing methods for semantic segmentation of microvascular decompression images also have some problems, such as the lack of feature information, incomplete target contours, and unclear target boundaries. Therefore, we added the FDB to the backbone network to further refine the features. At the same time, the ASPP module is added to the decoder structure, and it is concatenated with the low-level feature map extracted from the backbone network to retain more feature information, which makes the boundary information of the target more complete and the semantic information clearer. Experimental results show that our method can obtain more feature information and clearer target boundaries, improve the accuracy of semantic segmentation of microvascular decompression images, and provide help for future intelligent medical treatment.

## Figures and Tables

**Table 1:** Classification number, name, and color table.

Number	Category Name	RGB Value	Color
1	cn5	(192, 192, 0)	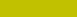
2	cn7	(0, 64, 0)	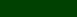
3	cn9	(64, 0, 128)	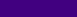
4	cn10	(64, 128, 128)	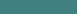
5	aica	(0, 0, 128)	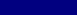
6	pica	(192, 0, 192)	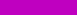
7	aica + cn7	(64, 0, 0)	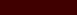
8	pica + cn7	(128, 0, 64)	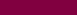
9	pv	(192, 128, 64)	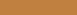

**Table 2:** Training parameters.

Parameters	Value	Parameter	Value
num clones	2	learning rate	0.05
iterations	41,000	momentum	0.9
atrous rates	6, 12, 18	weight decay	0.00004
output stride	16	crop size	512 × 512
decoder output stride	4	batch size	4

**Table 3:** MIoU value of microvascular decompression image dataset during microvascular decompression.

Model	Train OS	Eval OS	MIoU%
DeepLabv3+	16	16	72.56
Our method	16	16	75.73

**Table 4:** Pre-class results on the test set. Our method outperforms existing approaches and achieves 75.73% in MIoU.

Methods	MIoU	cn5	cn7	cn9	cn10	aica	pica	aica + cn7	pica + cn7	pv
U-Net	73.93	**81.81**	71.89	**77.56**	**81.88**	63.47	73.3	76.54	**87.76**	51.17
PSPNet	68.57	80.69	76.96	63.62	72.81	58.65	68.89	74.8	86.55	34.19
DeepLabv3+	72.56	81.33	77.87	65.62	69.58	68.2	68.52	75.29	84.6	**62.07**
DANet	69.49	78.81	71.38	69.97	72.37	55.39	67.2	74.95	85.49	49.84
FastFCN	70.21	78.13	76.18	74.59	73.83	57.35	71.92	76.22	85.0	38.67
Our method	**75.73**	81.07	**82.8**	74.48	79.18	**70.8**	**74.06**	**76.58**	86.58	56.06

**Table 5:** MIoU values in different situations.

Encoder	Our Encoder	Decoder	Our Decoder	MIoU%
	√	√		74.43
√			√	74.57
	√		√	75.73

## Data Availability

Restrictions apply to the availability of these data. Data was obtained from the First Hospital of Jilin University and are available from the authors with the permission of the First Hospital of Jilin University.
